# Massive Health Record Breaches Evidenced by the Office for Civil Rights Data

**Published:** 2019-02

**Authors:** Waldemar W. KOCZKODAJ, Jolanta MASIAK, Mirosław MAZUREK, Dominik STRZAŁKA, Pavel F. ZABRODSKII

**Affiliations:** 1. Computer Sciences, Laurentian University, Sudbury, Canada; 2. Independent Neurophysiological Unit, Department of Psychiatry, Medical University of Lublin, Lublin, Poland; 3. Faculty of Electrical and Computer Engineering, Rzeszów University of Technology, Al. Powstańców Warszawy 12, 35-959 Rzeszów, Poland; 4. Saratov Medical University “REAVIZ”, Saratov, Russian Federation

**Keywords:** Health, Civil rights, Health data breaches

## Abstract

**Background:**

Using data collected by the Office for Civil Rights, Department of Health and Human Services (HHS), over half of the population in the USA might have been affected by security breaches since Oct 2009. This study provided analysis of the data, presenting the numbers of individuals affected in one breach and the number of breaches.

**Methods:**

Statistical analysis has been conducted with visualizations. Visualizations include categorized histograms and tables. Histograms are presented as bar charts with categories: location and breach type. Tables show case counts (across top 10 breaches and those with more than one million stolen records) in successive years and covered entity types. All statistics were calculated with the use of package R. Analyzed data were collected from Oct 2009 till Jun 2017.

**Results:**

This study presents evidence of health data breaches taking place at an unprecedented level. Medical records of at least 173 million of people, gathered since Oct 2009, have been breached and might have adversely influenced over half of the population in the USA.

**Conclusion:**

Results of this study are expected to motivate public care authorities to develop similar laws and regulations as the USA while striving for better law enforcement. It takes a considerable amount of time to educate public and it takes substantial financial resources to prevent data breaches.

## Introduction

The main goal of this study was to send a strong signal to Iran and the neighboring countries to pay more attention to security and privacy issues before it comes to the level of disaster as demonstrated by the reliable statistics below.

Analysis of medical records is an ongoing problem. There has been a steady increase in security breaches of data processing systems reported in some studies (1). However, our study reports a problem of monumental proportions. Medical errors are the third leading cause of death in the USA. The need for patients to protect themselves and their families from harm, and for hospitals to make patient safety a priority is evident. Many hospitals are making headway in addressing errors, accidents, injuries and infections that kill or hurt patients, but overall progress is not impressive (2, 3). For many years there has been a steady increase in the number of security breaches of data processing systems (1, 4–6). With the rapid development of computer networks, the network is confronting a growing number of threats. Therefore, it is very important to assess the risks to the network information system. Internet application technologies, such as cloud computing and cloud storage, have drastically changed peoples’ lives. Websites contain vast amounts of personal privacy information. In order to protect this information, network security technologies, such as database protection and data encryption, attract many researchers. Cybercriminals′ attacks focus not only on obtaining medical data, but also on other database, communication and production systems (7). The challenge is to use new methods for fighting terrorism and to detect and prevent security breaches e.g. Data Mining, Semantic Web and Advanced Information Technologies (8–10). The most serious problems concerning web vulnerability are e-mail address and network database leakages. These leakages have many causes. For example, malicious users can steal database contents, taking advantage of mistakes made by programmers and administrators (11).

The security of electronic health records (EHR) is critical (12). The important role of electronic health records should influence the development of essential infrastructures for safety and privacy preservation. The technology used must be accepted by users, inexpensive and simple enough while less vulnerable to changes and data breaches. Increasing healthcare cost due to trends such as demographic and epidemiologic transition and uncontrolled increase in using new technologies in health care is one of the most important threats that the health system will be facing (13). Our empirical study shows that the confidentiality of electronic health records (EHR) is breached at an unprecedented level. As much as half of the US population may have electronic health records compromised and it calls for a social action.

## Methods

The methods used in this study were simple statistics driven by the discovery of data collected by the Office for Civil Rights, Department of Health and Human Services (HHS). Discovery is regarded as the act of detecting something previously unrecognized as meaningful. In our case, it was the extent of health data breaches in USA gathered from October 2009 till June 2017 by HHS. Access to the analyzed data is public on the web-site. We also use data taken from the Internet Live Stats (www.internetlivestats.com/), which is a part of the Real Time Statistics Project. The data were stored in one Excel worksheet which was subsequently analyzed by basic statistics of R (a statistical open source system). In addition, two statistical systems: Statistica and Origin were used to extract data, produce presented tables, histograms, and visualizations.

Data analysis was conducted by a three-step approach. In the first step, we extracted data only related to health record breaches. The next step was to divide the extracted health data breaches into two main categories: the number of individuals affected (NIA) and the number of breaches (NB). Step three is referred to as the detailed analysis of NIA and NB. Functions included in the R package divided the extracted data into: (i) five main categories of breaches location: Business Associate (BA); Health Plan (HP); Healthcare Clearing House (HCH); Healthcare Provider (HPr), and Uncategorised (UN); (ii) seven categories related to the type of breach: Hacking/IT Incident (A); Improper Disposal (B); Loss (C); Other (D); Theft (E); Unauthorized Access/Disclosure (F); Unknown (G). This extraction was possible thanks to the preliminary data categorization available from original data sources. It was done in order to highlight only those cases, which were important for the analyzed topic. Histograms (related to NIA and NB) were produced and presented as bar charts for categories obtained in step 2.

## Results

### The general data breaches situation

**Fig. 1:**
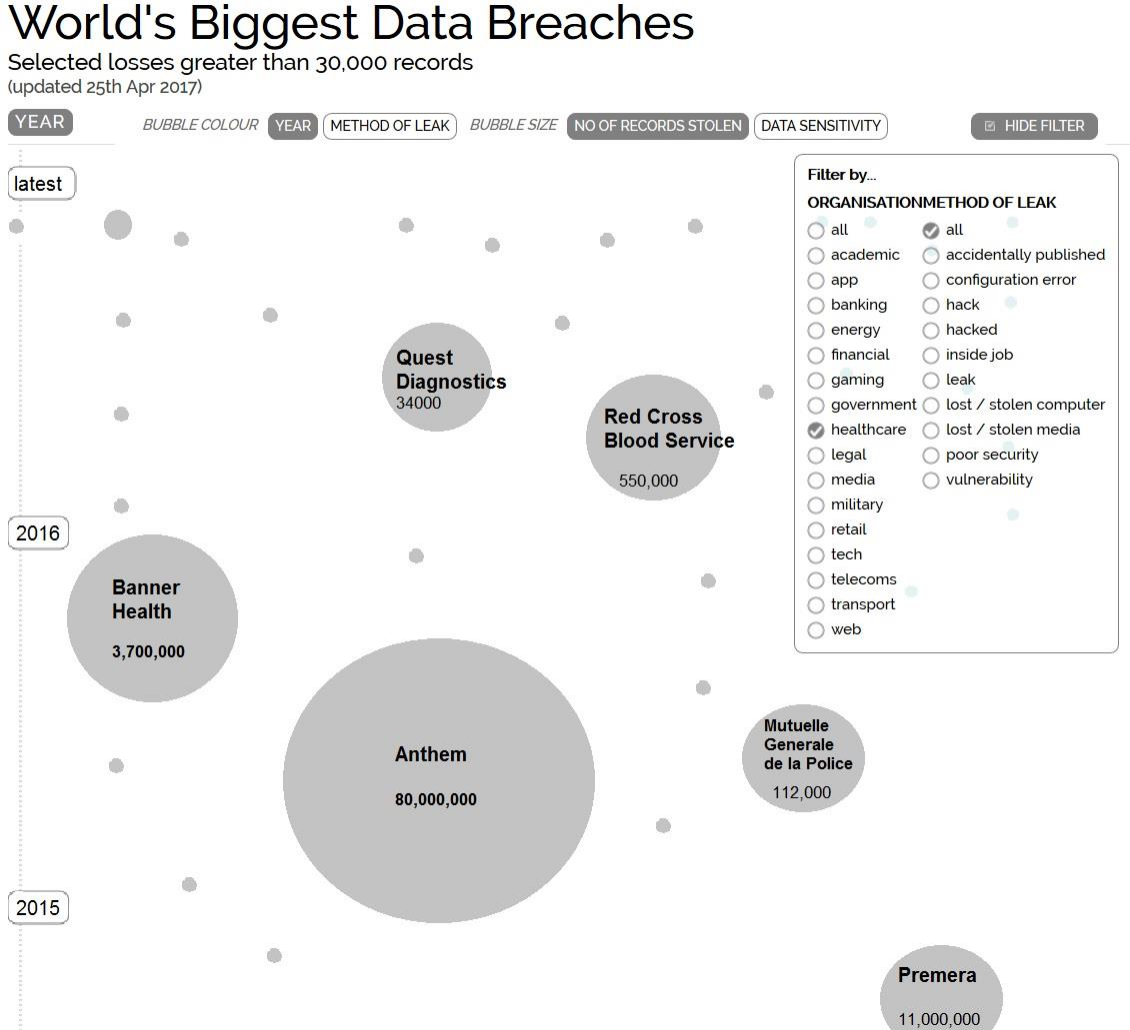
World’s biggest data breaches filtered by healthcare category

Let us see how the general (not necessary health care situation) looks like. The disastrous data breaches are nicely visualized in (14). Unfortunately, they rely on public media as the source hence not acceptable for research. The portal presents infographics from well-known sources such as Routers, Consumer News and Business Channel (CNBC), National Broadcasting Company (NBC), New York Times (NYTimes), Cable News Network (CNN), Guardian, and many industry portals PC World, Computer Weekly, etc. The portal’s mission is to distill the world’s data, information and knowledge into impressive and useful graphics and diagrams. They base all graphics and visualisations on facts and data. The top 10 lost records are shown in Table 1. Table 2 and Fig. 1 show the top 10 data of healthcare records stolen.

**Table 1:** Top 10 data breaches in the lost records category

	***Year***	***Entity***	***Records lost***	***Organisation***
1	2017	River City Media	1,370,000,000	Web
2	2013	Yahoo	1,000,000,000	Web
3	2014	Yahoo	500,000,000	Web
4	2017	Friend Finder Network	412,000,000	Web
5	2012	Court Ventures	200,000,000	Financial
6	2015	Voter Database	191,000,000	Govermment
7	2016	My Space	164,000,000	Web
8	2012	Massive Amer.bsn hack	160,000,000	Financial
9	2009	Heartland	130,000,000	Financial
10	2012	Linkedln	117,000,000	Web

**Table 2:** Top 10 data of healthcare records stolen

	**Year**	**Entity**	**Number of records stolen**	**Records lost**	**Method of leak**	**Data Information source**
1	2016	Anthem	130,000	80,000,000	Hacked	Zdnet.com
2	2015	Premera	150,000	11,000,000	Hacked	Infoworld.com
3	2014	Twitch.tv	150,000	10,000,000	Web	The State, HHS
4	2011	NHS	160,000	8,300,000	Lost/stolen	RowStory.com
5	2012	South Carolina Gov.	180,000	6,400,000	Inside job	Washingtonpost
6	2014	Health Systems	250,000	4,500,000	Hacked	Forbes
7	2011	Sutter Medical Found.	250,000	4,243,434	Lost/stolen	Cnet.com
8	2013	Advocate Med. Group	300,000	4,000,000	Lost/stolen	KoreaTimes
9	2016	Banner Health	500,000	3,700,000	Hacked	Wired.co.uk
10	2011	Health Net - IBM	1,000,000	1,900,000	Lost/stolen	Cnet.com

Our privacy is at stake. We have the right to privacy and high expectations that our medical records are protected, but are they really? Our findings have stunned us to the extent that we needed to assess whether or not they could be regarded as credible. However, the reliability of the data source (number of security of data breaches recorded by the US government agency) and its availability (posted for everyone to access and verify our finding) as well as the importance of data ( https://ocrportal.hhs.gov/ocr/breach/breach_report.jsf ) have convinced us that publishing our findings is in the public interest.

The legal term: the number of individuals affected (NIA) is carefully chosen by the governmental agency and it does not reflect a simple fact that it may be you or someone close to you. For sure, it is a human being. His/her life may be ruined or even shortened if he/she has an illness or condition which may not be wise for him/her to disclose.

The total NIA is 173,627,498 (∼173.6 million people) between Oct 2009 and Jun 2017. On average, one data breach takes place every 1.5 day. The total number of individuals affected in 33 data breaches in March 2015 is 91,775,871 (over 91 million). In time series analysis of truncated HHS data we observe a slow upward trend of number of breaches (NB). Table 3 illustrates the total NIA and total NB in years (only 3 months in 2009 and 2017).

**Table 3:** Number of individuals affected and number of breaches years (only last 3 months in 2009 and the first 6 months of 2017 are recorded)

	***Year***	***Number of individuals affected***	***Number of breaches***
1	2009	134,773	18
2	2010	5,932,276	199
3	2011	13,150,298	195
4	2012	2,808,042	201
5	2013	6,939,276	265
6	2014	12,682,073	289
7	2015	113,267,174	267
8	2016	16,655,952	328
9	2017	1,828,956	101
10	total	173,627,498	1,957

The development of the market for stolen data and related hacking skills indicate that the business of perpetrators in the health care sector is growing. Actions of perpetrators are becoming more and more aggressive and it is not uncommon for them to even use online ads and social media for recruiting health care insiders having access to valuable data. Upon stealing a cache of medical records, it is likely perpetrators have to analyze the data, and perhaps cross-reference it with data from other sources before lucrative fraud, theft, extortion, or blackmail opportunities can be identified (15). Financial data, therefore, still present a faster, more attractive return-on-investment opportunity for perpetrators.

An important issue to resolve is the effect of Internet security breach announcements on market value. Any information that leaks into the network poses a major threat to the capital markets, companies and may be a source of speculation on the stock markets.

### Security breach data regulations of the Office for Civil Rights, Department of Health and Human Services (HHS) in USA

The security breach data has been collected by the Office for Civil Rights, HHS in the USA. The data collection is involuntary and regulated by Section 13402 of the Health Information Technology for Economic and Clinical Health (HITECH) Act which is a part of the American Recovery and Reinvestment Act of 2009 (ARRA). ARRA was enacted on Feb 17, 2009 by requiring HHS to issue interim final regulations within 180 d. Entities under the Health Insurance Portability and Accountability Act of 1996 (HIPAA) and their business associates are required to provide notification in the case of breaches of health data. HHS is requested to update its guidance specifying the technologies and methodologies that render protected health information unusable, unreadable, or indecipherable to unauthorized individuals.

According to (16):

The HITECH Act, Title XIII of Division A and Title IV of Division B of the American Recovery and Reinvestment Act of 2009 (ARRA) (in USA Public Law 111–5), was enacted on Feb17, 2009. Subtitle D of Division A of the HITECH Act (the Act), entitled ”Privacy”, among other provisions, requires the HHS or the Department to issue interim final regulations for breach notification by covered entities subject to the Administrative Simplification provisions of the HIPAA (in USA Public Law 104–191) and their business associates.

Section 13402 of the HITECH Act regulates the breach notification process. It applies to HIPAA covered entities and their business associates that access, maintain, retain, modify, record, store, destroy, or otherwise hold, use, or disclose unsecured protected health information. The Act defines ”covered entity”, ”business associate”, and ”protected health information” used in the HIPAA Administrative Simplification regulations (45 CFR parts 160, 162, and 164; Title 45: Public Welfare in Code of Federal Regulations; parts: 160 - General Administrative Requirements, 162 - Administrative Requirements, 164 - Security and Privacy) at §160.103. Under the HIPAA Rules, a covered entity is:

- A health plan,
- Health care clearinghouse,
- Health care provider that transmits any health information electronically in connection with a covered transaction, such as submitting health care claims to a health plan.

There are 12 top breaches with NIA for entities with ″healthcare″ in their name higher than 1,000,000 (Table 4).

**Table 4:** Breaches with NIA 1,000,000+ for entities with “healthcare” in their name

***State***	***Covered.Entity.Type***	***Breach.Submission.Date***	***Individuals.Affected***
IN	Health Plan	03/13/2015	78,800,000
WA	Health Plan	03/17/2015	11,000,000
NY	Health Plan	09/09/2015	10,000,000
CA	Healthcare Provider	07/17/2015	4,500,000
IL	Healthcare Provider	08/23/2013	4,029,530
AZ	Healthcare Provider	08/03/2016	3,620,000
FL	Healthcare Provider	03/04/2016	2,213,597
FL	Health Plan	06/03/2010	1,220,000
MD	Health Plan	05/20/2015	1,100,000
MT	Health Plan	07/07/2014	1,062,509
FL	Healthcare Provider	10/07/2011	1,055,489
TN	Health Plan	11/01/2010	1,023,209
		Total	119,624,334

A business associate, defined by the HIPAA Rules, is a person or service performing functions or activities on behalf of a covered entity. It involves the use or disclosure of individually identifiable health information. Business associates include third party administrators or pharmacy benefit managers involved in health plans, claims processing. Business associated may work in billing companies, transcription companies. They may also provide legal, actuarial, accounting, management, or administrative services for covered entities and who require access to protected health data.

According to (16):

The HIPAA Rules define ”protected health information” as the individually identifiable health information held or transmitted in any form or medium by these HIPAA covered entities and business associates, subject to certain limited exceptions.

The HITECH Act requires HIPAA covered entities to provide notification to affected individuals and to the Secretary of HHS following the discovery of an unsecured protected health information breach. The unsecured protected health information is the official Act term. In addition, in some cases, the Act requires covered entities to provide notification about breaches to the media. In case of a breach of unsecured protected health information at or by a business associate of a covered entity, the Act requires the business associate to notify the covered entity of the breach. Finally, the Act requires the Secretary to post on an HHS Web site a list of covered entities that experience breaches of unsecured protected health information involving more than 500 individuals.

Section number 13400 (1) of the HITECH Act defines ”breach” to mean, generally, the unauthorized acquisition, access, use, or disclosure of protected health information which compromises the security or privacy of such information. The Act provides exceptions to this definition to encompass disclosures where the recipient of the information would not reasonably have been able to retain the information, certain unintentional acquisition, access, or use of information by employees or persons acting under the authority of a covered entity or business associate, as well as certain inadvertent disclosures among persons similarly authorized to access protected health information at a business associate or covered entity.

Further, section number 13402(h) of the HITECH Act defines ”unsecured protected health information” as ”protected health information that is not secured through the use of a technology or methodology specified by the Secretary in guidance” and provides that the guidance specify the technologies and methodologies that render protected health information unusable, unreadable, or indecipherable to unauthorized individuals. Covered entities and business associates that implement the specified technologies and methodologies with respect to protected health information are not required to provide notifications in the event of a breach of such information -that is, the information is not considered “unsecured” in such cases.

Section number 13407 (f) (3) of the HITECH Act stipulates that “unsecured personal health records” (UPHR) are “identifiable health information” that is not protected through the use of a technology or methodology specified by the Secretary of HHS. Section 13402 of the Act requires breach notification following the discovery of a breach of unsecured protected health information.

### Health data security problems, collected data, and methodology

*Internet Live Stats* (*www.internetlivestats.com/*) is a part of the Real Time Statistics Project (Worldometers and 7 Billion World). The following global data website Worldometers has been voted as the best on-line reference website by the American Library Association (ALA). Their statistics are referenced in over 400 published books and more than 150 professional journal articles. It allows us to watch the Internet statistics as they change in real time. We also can monitor social media usage: the number of Internet users, websites, blog posts, Facebook, Google+, Twitter, and Pinterest. It also provides other useful information, for example, about hacked websites. This is not only the matter of the Internet’s growth but also a very serious issue, namely, Internet security and, in general, IT systems security (17). O’Connor (2011) wrote: “half of states have no statutes addressing non-disclosure of personally identifiable health information generally held by public health agencies” (18).

The real explosion of Internet use, is on the other hand attributed to two milestones:

- The initial release of Mosaic (web browser) on Jan 23, 1993,
- The National Science Foundation (NSF) lifted the ban of commercial use in 1991.

In 1995, the NSF began charging a fee for registering domain names and registered 120,000 domain names. This number grew to over 2 million in three years and the NSF no longer controlled the Internet. Since then, the Internet has become the most convenient, fastest, and cheapest way to access data including health records. Whenever there exists a possibility of having direct or indirect access to some resources (not necessarily protected), there always exists enticement to breach these resources and make use of them for financial benefit.

**Fig. 2:**
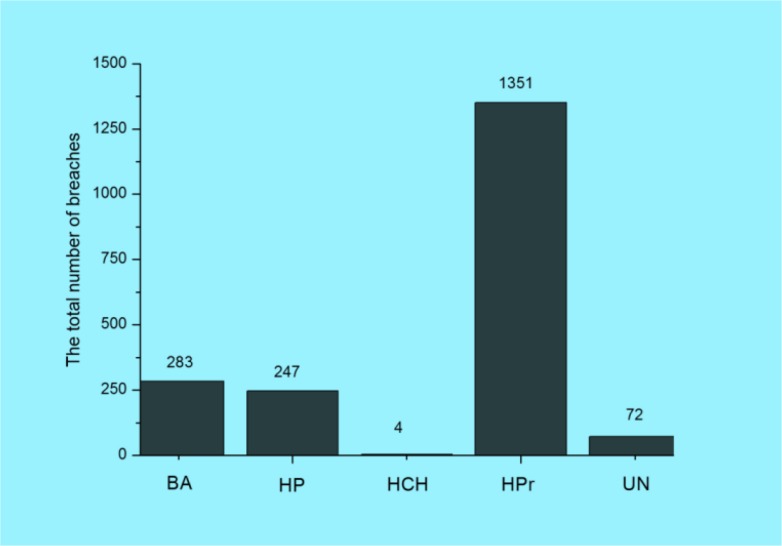
The total number of breaches by the type of breaches location

**Fig. 3:**
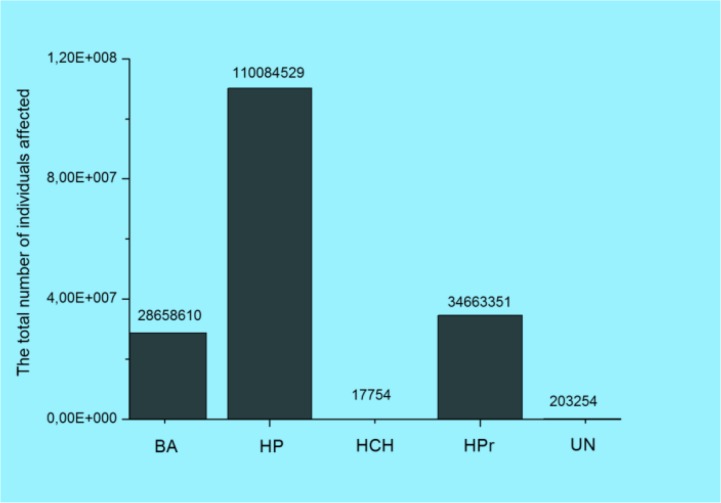
The total number of individuals affected by the type of breach location

The analyzed data were grouped for five main categories of location of breaches: Business Associate (BA); Health Plan (HP); Healthcare Clearing House (HCH); Healthcare Provider (HPr), and Uncategorised (UN). Figs. 2 and 3 demonstrate total number of breaches and individuals affected.

The average time between health data breaches is approximately 1.5 d (since Oct 2009, the initial date of recording). In one month (Nov 2016), there were 38 data breaches with a total number of 776,797 individuals affected. The monthly average of number of individuals affected (NIA) is 1,907,365 (nearly 2 million individuals).

The bar chart in Fig

**Fig. 4:**
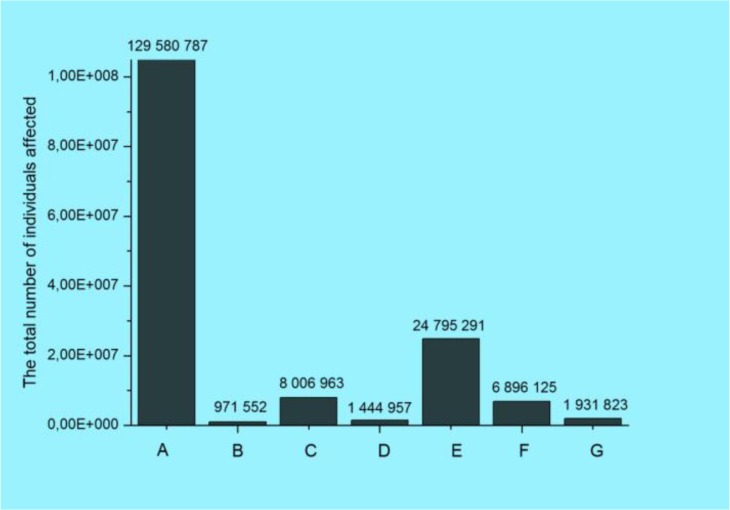
The total number of individuals affected by the type of breaches

**Fig. 4** shows the total number of individuals affected (NIA) in one data breach by the type of breach (category): Hacking/IT Incident (A); Improper Disposal (B); Loss (C); Other (D); Theft (E); Unauthorized Access/Disclosure (F); Unknown (G). The official categorization in Fig.

**Fig. 4** has fundamental flaws. It indicates nearly 2,000,000 NIA as unknown. ∼6,900,000 NIA are categorized as ”unauthorized” implying that approx. 130,000,000” hacking/IT incidents” may be authorized. Although it may be taken for a case of black humor, it serves as an evidence that even basic breach terminology has not been developed yet. The bar chart in Fig.

**Fig. 5:**
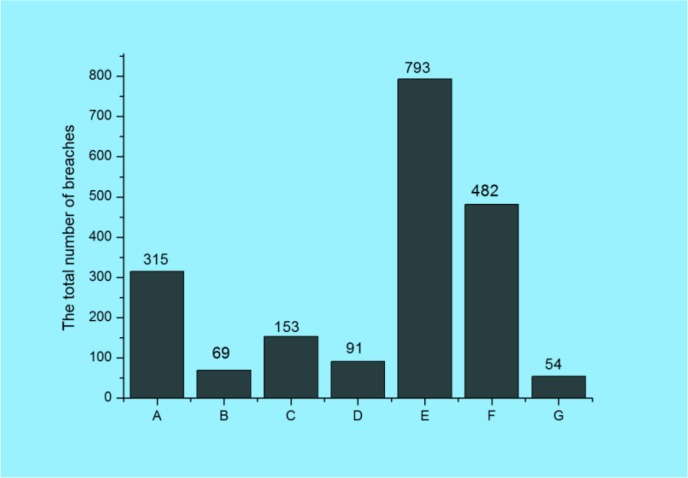
The total number of breaches by the type of breaches

**Fig. 5** shows the total number of breaches (NB) by type of breach: by type of breach. The largest numbers of individuals affected (NIA) belong in the theft category, which represents 40.5% of all number of breaches. The lowest numbers of breaches are in the Unknown category and reached only 2.75%.

## Discussion

The HHS posted data contain information about NIA in one breach and the total *NB*. Since Oct of 2009, the total NIA is 173,627,498. The total NB is 1,957. NB is not rapidly growing but the amount of stolen data is growing. It is an ongoing struggle. Hopefully, it may be won, but at high cost since the practice of subcontracting low paid consulting offices may not be an acceptable solution for processing highly sensitive medical records. Selling personal information such as social insurance numbers, telephone number, address, and birth date is so frequently practiced that the law enforcement is practically powerless since perpetrators usually operate outside jurisdictions or use ”darknet” and are virtually untraceable. One may wonder who needs a million of medical records. Unfortunately perpetrators do not post their clients on the Internet but, in all likeliness, big corporations (especially live insurance industry) may benefit from it. Perpetrators focus not only on obtaining medical data, but also on other database, communication and production systems.

The development of the market for stolen data and related hacking skills indicate that the business of cybercrime in the healthcare sector is growing. The researchers also observed brazen efforts by cybercriminals, through online ads and social media, to recruit into their ranks healthcare industry insiders with access to valuable information. The findings suggest financial account data continues to be easier to monetize than personal medical data, which could require an investment that financial payment data does not require. Upon stealing a cache of medical records, it is likely cybercriminals have to analyze the data, and perhaps cross-reference it with data from other sources before lucrative fraud, theft, extortion, or blackmail opportunities can be identified. Financial data, therefore, still presents a faster, more attractive return-on-investment opportunity for cybercriminals (19–21). An important issue to solve is the effect of internet security breach announcements on market value (22). Any information that leaks into the network poses a major threat to the capital markets, companies and may be a source of speculation on the stock markets. Capital markets react very quickly to breached firms and internet security developers. In today’s world applying correct security policies and tools is necessary.

Stolen medical records are sold in bulk on the black market for as little as $10 per person. Social insurance numbers (needed to identity the theft) of individuals may ”go” for hundreds of dollars on the black market. Selling personal information such as telephone number, address, and birth date is so frequently practiced that the law enforcement is practically powerless since perpetrators usually operate outside jurisdictions or use ”darknet” and are virtually untraceable. One may wonder who needs a million of medical records. Unfortunately perpetrators do not post their clients on the Internet but, in all likeliness, big corporations (especially live insurance industry) may benefit from it. Criminal attacks focus not only on obtaining medical data, but also on other database, communication and production systems. It is important to improve the awareness of public about the seriousness of the problem. More financial resources should be allocated for development of new methods. Stricter laws should be passed.

## Conclusion

Thanks to data collected by the Office for Civil Rights, Department of Health and Human Services (HHS), it was possible to present the credible evidence about health data breaches which accounts for over half the population in the USA. This study presents evidence of health data breaches taking place at an unprecedented level. This study will motivate other countries to develop similar laws and regulations as the USA while striving for with a better law enforcement.

We hope to bring to the attention of Iran and the neighboring countries that potential privacy issues should be regarded as a major concern. It takes considerable time to educate public and it takes substantial financial resources to prevent data breaches.

## Ethical considerations

Ethical issues (Including plagiarism, informed consent, misconduct, data fabrication and/or falsification, double publication and/or submission, redundancy, etc.) have been completely observed by the authors.
